# Femoral condyle configuration and its impact on anterior cruciate ligament reconstruction

**DOI:** 10.3233/THC-220640

**Published:** 2023-09-15

**Authors:** Lena Riemer, Jens Dargel, Peter Schäferhoff, Peer Eysel, Thomas Steimel, Sönke Reineck, David Grevenstein

**Affiliations:** a Department for Orthopaedic and Trauma Surgery, Faculty of Medicine, University of Cologne, Cologne, Germany; b Department for Orthopaedic Surgery, St. Josefs-Hospital Wiesbaden, Wiesbaden, Germany; c Department for Orthopaedic Surgery, ATOS MediaPark Klinik, Cologne, Germany; d Radiologie im Mediapark, Cologne, Germany

**Keywords:** Anterior cruciate ligament, ACL, ACL-tear, knee, femoral condyle, knee anatomy

## Abstract

**BACKGROUND:**

Rupture of the anterior cruciate ligament (ACL) is one of the most common knee injuries and has substantial impact on knee function. Beside primary ruptures, an increasing number of re-(re-)ruptures occur, representing a therapeutical challenge for the treating surgeon. Several risk factors for re-ruptures have been previously identified, including an increased tibial slope.

**OBJECTIVE:**

In this study, we investigated the effect of femoral condyle configuration on ACL-ruptures and re-ruptures.

**METHODS:**

*In-vivo* magnetic resonance imaging scans of three different groups of patients were compared. Group 1 included patients with an intact ACL on both sides, group 2 included patients with primary, unilateral ACL-rupture, while group 3 included patients with an ACL-re-rupture or re-(re-)rupture. Fourteen different variables were obtained and analyzed regarding their impact on ACL-re-(re-)rupture.

**RESULTS:**

Overall, 334 knees were investigated. Our data allowed us to define parameters to identify anatomical configurations of bones associated with an increased risk of ACL-re-rupture. Our results show, that patients with ACL-re-rupture show increased radii of the extension facet of the lateral femoral condyle (p< 0.001) as well as of the extension facet of the medial femoral condyle (p< 0.001).

**CONCLUSION:**

We conclude that a spherical femoral condyle form does influence the clinical outcome after ACL-reconstruction.

## 1. Introduction

Rupture of the anterior cruciate ligament (ACL) is one of the most common knee injuries, with an incidence between 32–38/100.000 person-years [1, 2, 3]; however, some studies report higher incidences of up to 68.6/100.000 person-years [4]. Contrasting with primary tears, re-rupture after ACL-reconstruction is quite rare, but still reaches a 5-year prevalence of around 6% [5]. ACL-ruptures are severe injuries, especially in patients performing sports-related activities. Despite reported return-to-sport rates to preinjury level of between 13–69% [6], treatment often requires surgery and strict rehabilitation. In particular, re-ruptures can be career-ending in professional athletes. Because of the relatively high incidence, especially in younger people, ACL-tears are socioeconomically relevant due to the need for surgeries and lengthy rehabilitation. Based on the abovementioned facts, ample research regarding surgical-techniques [7], graft-choice [8, 9], and post-surgery rehabilitation [10] has been published. While the surgical technique was the main topic in the beginning of ACL research, arthroscopically assisted ACL-reconstruction represents the golden standard today. Today, several grafts are available for ACL-reconstruction, whereby the autologous semitendinosus-, patella- and quadriceps-tendon can be considered as equally good, with several pros and cons for each graft [11]. In theory, preconditioned grafts should be advantageous [12]. Moreover, drilling of the femoral tunnel, which was performed transtibial in the early years, is commonly performed through the anteromedial portal now [13]. Additionally, possible risk factors for ACL-rupture have become a popular research target. Several authors have investigated the influence of anatomical configurations and structures of the knee on ACL-function and stability. Park et al. found that in both males and females with ACL-rupture, the notch width (NW) appeared smaller on magnetic resonance imaging (MRI) scans, compared to controls [14]. Similar results were reported by two other studies which found an anterior outlet-stenosis of the notch on computed tomography (CT) scans that showed significant association with ACL-tears [15, 16]. Besides the notch, the influence of several other anatomical variants of various knee structures on ACL-stability have been investigated. A follow-up study of 100 patients with ACL-tears reported that conservative treatment of ACL-tears led to less satisfying results in patients with a more spherical shape of the femoral condyles [17]. It has been postulated that patients who underwent surgery reached higher subjective scores when they had a smaller intercondylar notch and a smaller width of the intercondylar eminence. Notably, conservatively treated patients scored better when a more pyramidal shaped notch was present [18]. Moreover, a significant smaller height of the lateral femur condyle seems to be a risk factor for ACL-rupture [19]. One study reported that a smaller tibial plateau length relative to the femur comes with a higher risk for ACL-tears. The authors argue that this could be one reason for the higher incidence of ACL-tears in females, since this anatomical relation was more often detected in female participants, even when no ACL-rupture was evident [20]. The morphometry of the femoral condyles seems to not only influence ACL-stability but also the risk for injuries associated with ACL-rupture. One study noted that an increased anteroposterior length of the medial/lateral femoral condyle relative to the medial/lateral tibial plateau was associated with an increased risk of meniscal lesions when combined with ACL-rupture [21]. Beside the notch and femoral condyles, tibial parameters also seem to influence ACL-stability. Another study reported that an increase of the posterior tibial slope (PTS) increased the risk for non-contact ACL-tears in females [22]. These results are supported by those of a meta-analysis, which analyzed the cumulative data of 12 studies. The analysis indicated that an increased medial and lateral tibial slope is associated with ACL-injuries [23]. Moreover, an increased PTS is associated with ACL-re-rupture [24]. This resulted in the option of tibial slope correction, which aims to reduce the posterior tibial slope and thereby to reduce the risk for ACL-re-tear [25]. Moreover, the combination of slope correction and varus correction could show reduced forces on ACL grafts in biomechanical analysis [26].

A study concluded that their results show no evidence that knee-stability can be derived from its radiographic surface geometry [27]. Another study also reported no significant differences between the shape of the femoral condyles in ACL-reconstructed knees compared to those with native ACL. One of the major limitations of this study, however, was that bilateral knees of the same patients were compared; thus, no relevant difference in the shape of the condyle between the knees of the same patient would be expected, even if one knee suffered from ACL-rupture while the other did not [28].

Clinically, a study reported that at three months post-trauma, a positive pivot-shift test is the strongest predictor of the future need for ACL-reconstruction in patients with ACL-rupture [29].

Against this background, we investigated whether different shapes of the femoral condyles are associated with a higher incidence of ACL-ruptures, re-ruptures, and re-(re-)ruptures. Papers recently focused on the PTS, but beside the PTS, several anatomical variants affect the ACL. Our hypothesis was that an elliptical shape of the femoral condyles offers better bony congruency and comes along with better intrinsic stability, thereby decreasing the risk for ACL-rupture or re-rupture after ACL-reconstruction.

## 2. Materials and methods

This study was performed in accord with the Declaration of Helsinki of 1964 and its later amendments Ethical approval was obtained from the local ethics committee (Approval number: VT- 21-1310).

**Figure 1.**
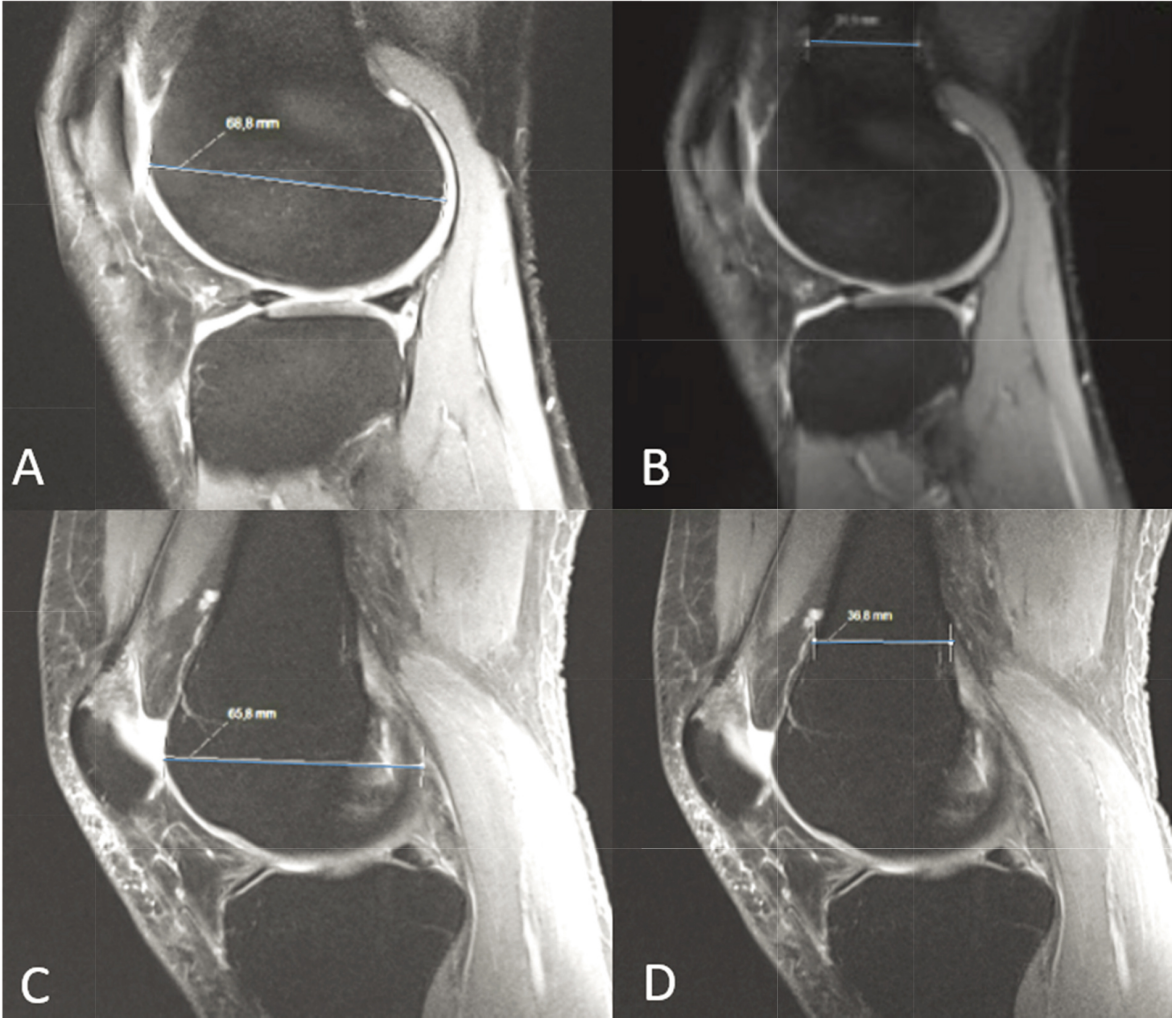
Variables 1–4 are shown in sections A–D. A: Variable 1 (depth of the lateral femoral condyle). B: Variable 2 (depth of the femoral shaft lateral). C: Variable 3 (depth of the medial femoral condyle). D: Variable 4 (depth of the medial femoral shaft).

**Figure 2.**
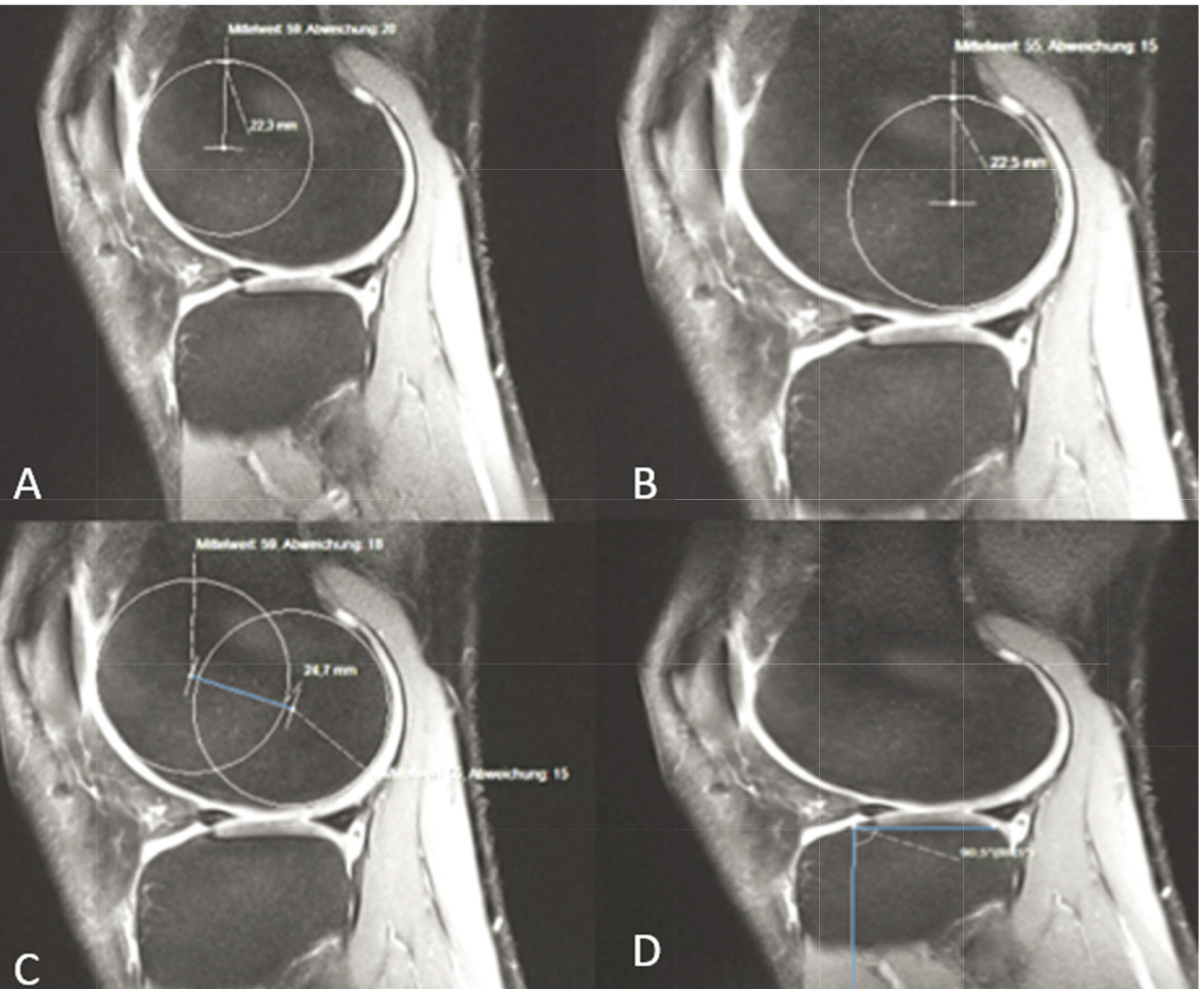
Variables 5–7 and 11 are shown in section A–D. A: Variable 5 (extension facet of the lateral femoral condyle). B: Variable 6 (flexion facet of the lateral femoral condyle). C: Variable 7 (distance between the circle-centers of the lateral femoral condyle). D: Variable 11 (angle between tibial plateau and tibial shaft lateral). Determination of the extension and flexion facet of the medial femoral condyle as well as the distance between the circle-centers was performed according to the demonstrated technique on the lateral side. Same applies to the determination of the medial tibial slope.

*In-vivo* MRI scans of three different patient groups were compared. Group 1 (n= 108, mean age 50 years, female n= 35, male n= 73) included patients with an intact ACL on both sides; group 2 (n= 118, mean age 32 years, female n= 33, male n= 85) included patients with primary, unilateral ACL-rupture; and group 3 (n= 108, mean age 34 years, female n= 32, male n= 76) consisted of patients with either an ACL re-rupture or re-(re-)rupture (mean age at primary rupture, 32 years; mean age at first re-rupture, 35 years; mean age at re-(re-)rupture, 39 years). Only one patient suffered from re-re-re-rupture (age: 50 years). There was no statistically significant difference regarding the gender between the three groups (p= 0.64). Exclusion criteria were additional ligamentous injuries in the MRI (e.g. MCL-, LCL- or PCL-lesions) and bucket handle tears, which required removal of large sections of the meniscus. Other lesions of the menisci and chondral lesions did not represent exclusion criteria. Patients in group 2 underwent arthroscopically assisted ACL-reconstruction using an semitendinosus autograft. Primary ACL-reconstruction in group 3 was performed in the same way, while revision surgery in group 3 was performed using a stripe of the patella-tendon with two adhering bone-blocks or a stripe of the quadriceps tendon with or without one adhering bone block. To analyze the individual surface geometry of the femoral condyles and tibial plateau, 14 standardized parameters were analyzed. The 12 measurements in the sagittal plane were as follows: depth of the lateral femoral condyle (value 1), depth of the femoral shaft lateral (value 2), depth of the medial femoral condyle (value 3), depth of the medial femoral shaft (value 4), extension facet of the lateral femoral condyle (value 5), flexion facet of the lateral femoral condyle (value 6), distance between the circle-centers of the lateral femoral condyle (value 7), extension facet of the medial femoral condyle (value 8), flexion facet of the medial femoral condyle (value 9), distance between the circle-centers of the medial femoral condyle (value 10), angle between tibial plateau and tibial shaft lateral (value 11), and angle between tibial plateau and tibial shaft medial (value 12). In the coronary plane, the height of the lateral (value 13) and medial intercondyle (value 14) were determined (Figs 1 and 2). We measured the angle between the tibial plateau and shaft medially and laterally, which is a 90${}^{\circ }$ angle minus the PTS. MRI scans were used, which allowed differentiation between the medial and the lateral PTS. Measurements were performed using IMPAX (Agfa Healthcare, Mortsel, Belgium).

Factors affecting image-quality were mainly related to image-quality due to moving artefacts, metal or implant artefacts, or massive joint effusion-including hemarthrosis, which can cause issues with the assessment of the anterior part of the condyles. Only images which allowed clear assessment of all relevant structures were used in this study.

Statistical analysis was performed using SPPS 22.0 (IBM, Armonk, New York, USA). A unifactorial analysis of variance (ANOVA) was used to compare mean values of the groups, while homogeneity of variances was tested using the Levene’s test. The values named above of 50 patients in group 1 were measured by three observers to test interobserver reliability. A p-value < 0.05 was considered statistically significant.

**Table 1:** Significant results regarding within group variables

Variables	Group	Significance (p-value)
1 (depth of the lateral femoral condyle)	3 > 1 and 2	p< 0.001
2 (depth of the femoral shaft lateral)	3 > 1 and 2	P= 0 < 0.001
3 (depth of the medial femoral condyle)	1 < 3	P= 0.002
4 (depth of the medial femoral shaft)	3 > 1 and 2	P= 0 < 0.001
5 (extension facet lateral femoral condyle)	1 > 2 and 3	p= 0.003 respectively p< 0.001
6 (flexion facet lateral femoral condyle)	2 < 1 and 3	p= 0.005 respectively p< 0.001
7 (distance between circle-centers lateral femoral condyle)	3 > 1 and 2	p< 0.001
8 (extension facet medial femoral condyle)	1 > 2 and 3	p= 0.006 respectively p< 0.001
9 (flexion facet medial femoral condyle)	1 > 2	p= 0.004
10 (distance between circle-centers medial femoral condyle)	3 > 2	p= 0.034
11 (Tibial slope lateral)	1 > 3	p= 0.028
13 (height area intercondylaris lateral)	3 < 1 and 2	p= 0.014 respectively p= 0.002
14 (height area intercondylaris medial)	3 < 1 and 2	p= 0.048 respectively p= 0.006
8:4	1 > 2 and 3	p= 0.002 respectively p< 0.001
5:2	1 > 2 and 3	p= 0.002 respectively p< 0.001

**Table 2:** Values for extension facet, depth of the femoral shaft and ACL-(re-)rupture index 1 and 2 within the three groups

	Extension facet medial	Extension facet lateral	Depth shaft medial	Depth shaft lateral	ACL-(re-) rupture- index 1: Extension	
facet						
medial						
÷						
Depth						
shaft						
medial	ACL-(re-) rupture- index 2: Extension facet lateral ÷ Depth					
shaft						
lateral						
Group 1 Intact ACL both sides	10.7 mm	11.8 mm	27.6 mm	30.9 mm	0.39	0.38
Group 2 Primary unilateral ACL-rupture	9.6 mm	10.2 mm	27.7 mm	30.6 mm	0.35	0.33
Group 3 ACL-re-(re-)rupture	9.3 mm	9.9 mm	30.9 mm	34.7 mm	0.3	0.29

## 3. Results

**Figure 3.**
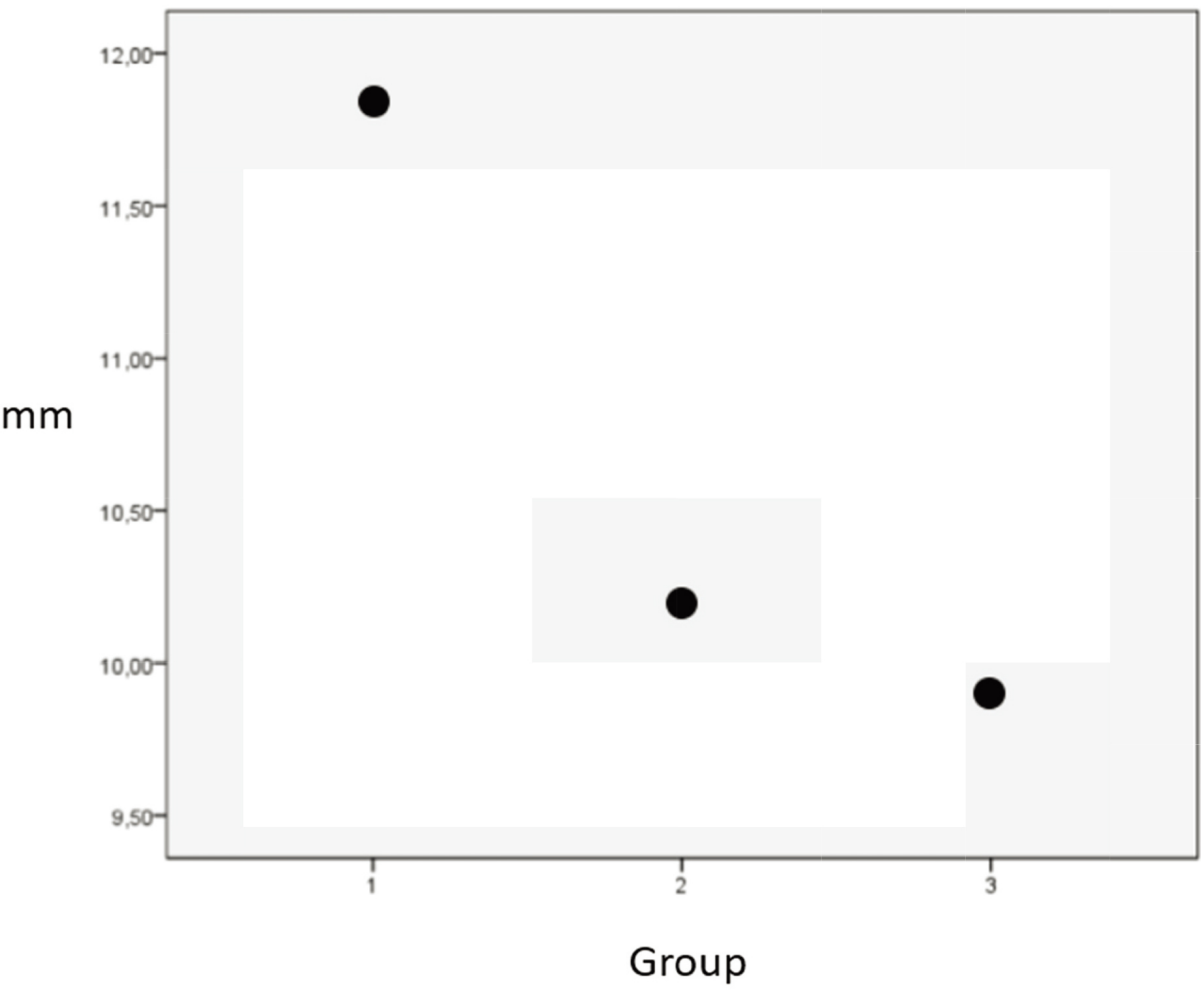
Size of the extension facet of the lateral femoral condyle in mm in groups 1 (intact ACL), 2 (unilateral ACL-rupture), and 3 (ACL-re-(re)rupture).

**Figure 4.**
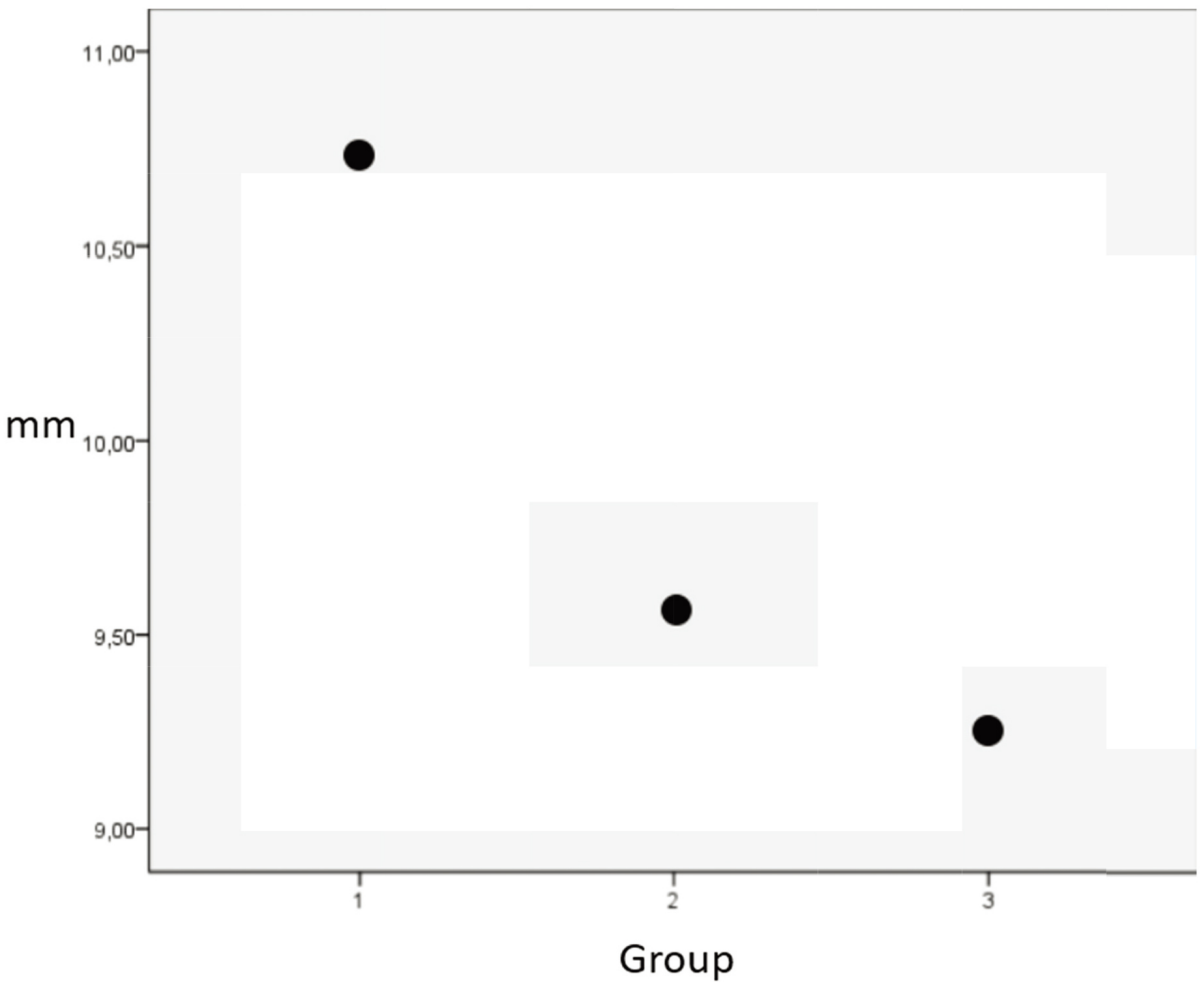
Size of the extension facet of the medial femoral condyle in mm in groups 1 (intact ACL), 2 (unilateral ACL rupture), and 3 (ACL-re-(re-)rupture).

In total, 334 MRI scans were analyzed. Sex distribution was homogenous in all three groups: approximately 1/3 females and 2/3 males in each group without significant differences regarding the distribution between the groups (p= 0.64). Distribution of the affected left and right knees was nearly equal in group 1 and 3; however, in group 2, more right knees (61%) were affected than left knees (39%), without significant difference (p= 0.052). There was no significant difference between the values of the three observers who measured 50 patients in group 1. Of the patients, 244 (73.1%) had an additional diagnosis, (e.g. lesion of the medial/lateral meniscus or partial resection of the medial/lateral meniscus in the past). Variables 2, 4, 5, 6, 8, and 9 showed non-homogenic variance; thus, Dunnett’s-F3-correction was performed to control for possible α-error. Variables 2 and 4 had significantly higher mean values in group 3 than in groups 1 and 2 (p< 0.001). Variable 5 showed a significantly higher mean value in group 1 than in groups 2 and 3 (1 vs. 2 p= 0.003, respectively; p< 0.001 comparing 1 vs. 3). Variable 6 showed a significantly smaller radius in group 2 than in groups 1 and 3 (2 vs. 1 p= 0.005 respectively p< 0.001 comparing 2 vs. 3). Variable 8 showed a significantly larger radius in group 1 than in groups 2 and 3 (1 vs. 2 p= 0.006 respectively p< 0.001 comparing 1 vs. 3). Variable 9 showed a significantly larger radius in group 1 than in group 2 (p= 0.004) (Figs 3 and 4); however, no significant difference was found between groups 1 and 3. For variables 1, 3, 7, 10, 11, 13, and 14, homogeneity of the variances could be proven and Bonferroni-correction for α-error was performed.

Variable 3 showed a significant difference when groups 1 and 3 were compared (p= 0.002), but no significant difference was noted when group 2 was compared to groups 1 and 3. Variable 1 showed a significantly larger distance in group 3 than in groups 1 and 2 (p< 0.001). Variables 13 and 14 were significant lower in group 3 than in groups 1 and 2 (variable 13: 3 vs. 1 p= 0.014 respectively p= 0.002 comparing groups 3 and 2; variable 14: p= 0.048 comparing groups 3 and 1 respectively p= 0.006 comparing groups 3 and 2). There was a significant difference between group 1 and 3 for variable 11 (p= 0.028). Variable 11 showed a significantly larger angle in group 1 than in group 3 (p= 0.028). Variable 12 did not show any significant difference between the three groups. Variable 10 showed a significantly larger distance in group 3 than in group 2 (p= 0.034); no significant difference was found between groups 1 and 3 (p= 0.087). Variable 7 showed a significantly larger distance in group 3 than in groups 1 and 2 (p< 0.001 for both). To calculate the proportion between variables 3 and 4, unifactorial ANOVA was used. The proportion showed homogenic variances; thus, Bonferroni correction was performed. The proportion between variables 3 and 4 showed a significantly lower value in group 3 than in groups 1 and 2 (3 vs. 1 p= 0.002 respectively p< 0.001 comparing group 3 vs 2). Variance analysis of the relationship between variables 1 and 2 revealed non-homogenous variances, which is why Dunnett’s-T3-α-correction was performed. The proportion between variable 1 and 2 showed a significantly lower mean value in group 3 (group 3 vs 1 p= 0.001 respectively p< 0.001 comparing group 3 vs 2). 

Unifactorial ANOVA was used to compare variables 5 and 6 and variables 8 and 9. For variables 5 and 6, group 3 showed a significantly smaller ratio compared to groups 1 and 2 (p< 0.001). Variables 8 and 9 revealed a significantly smaller ratio in group 3 than in groups 1 and 2 (p< 0.001).

ANOVA of the ratio of variables 8/4 revealed non-homogenous distribution; thus, a Games-Howell correction was performed. This was repeated for the ratio of variables 5/2. Group 1 showed significantly higher mean values than group 2 and 3 (p= 0.002 respectively p< 0.001) for the 8/4 ratio. Group 2 showed a significantly higher ratio than group 3 (p< 0.001).

Regarding the variable 5/2 ratio, group 1 showed significantly higher values than groups 2 and 3 (1 vs. 2 p= 0.002 and p< 0.001 comparing 1 vs. 3), and group 2 revealed a significantly higher ratio than group 3 (p< 0.001).

Variables 13 and 14 were compared using a t-test. Results showed a significantly higher mean value of the medial eminentia than the lateral eminentia (p< 0.001). Comparison of values for variable 11 and 12 showed significantly higher mean values for the lateral angle (value 11) (p< 0.001).

The risk for an ACL-re-rupture increases with the decrease of the radius of the extension facet of the lateral femoral condyle (OR = 0.778, CI = [0.691; 0.876], p< 0.001). The same can be predicted for the extension facet of the medial femoral condyle (OR = 0.783, CI = [0.695; 0.883], p< 0.001). 

## 4. Discussion

In this study, we measured 14 anatomical variables around the knee joint in MRI scans from patients with intact ACL (group 1); patients with primary, unilateral ACL-rupture (group 2); and patients with ACL-re-rupture or ACL-re-(re-)rupture (group 3). Two variables showed a significant difference between patients with an intact ACL and those with ruptured or re-(re-)ruptured ACLs: the extension facets of the lateral and medial femoral condyles. Group 1 showed a significantly larger radius of the medial and lateral extension facets compared to groups 2 and 3. In terms of 6 of the 14 variables, patients with ACL-re-rupture differed significantly from those in the groups with an intact ACL or an ACL-rupture. Patients in group 3 show a smaller radius of the extension facet of the lateral and medial femoral condyle. Moreover, in patients with ACL-re-rupture, the tuberculi of the eminentia are lower, and the distance between both centers of the circles in the lateral femoral condyle is larger, probably resulting in a longer running surface of the lateral condyle. Moreover, four ratios, which are significantly smaller in patients with ACL-re-rupture, can be calculated. The ratios are as follows: depth of the femoral condyle/femoral shaft both -medial and lateral- and the ratios of extension facet/flexion facet -both medial and lateral.

Some of the variables described in this study have been investigated by previous studies. An increase of the PTS is a well-studied risk factor for ACL-rupture [22, 23, 24]. Kapandji et al. reported that the medial tuberculum intercondylaris is higher than the lateral [30]. This is in line with our data. Kostogiannis et al. reported that a spherical configuration of the femoral condyles is an indicator that ACL-reconstruction will likely be necessary in case of ACL-rupture [31]. In our study, a spherical configuration of the femoral condyle was determined by lower values regarding the variables 1, 3, 7, 10 and the ratios 1/2 and 3/4.

Eggerding et al. postulated that a spherically shaped femoral condyle does not influence the clinical result after ACL-reconstruction [18]. Our results do not support these findings. All variables described above, which describe a spherical femoral condyle, show significant differences in patients with ACL-re-rupture compared to at least group 1, 2 or both. Thus, we conclude that a spherical form of the femoral condyle could influence the clinical outcome after ACL-reconstruction.

Several studies have noted that an increase of the tibial slope is associated with a higher risk for ACL-rupture or ACL-(re-)(re-)rupture [22, 23, 24, 32]. Normally, PTS is measured using radiography. However, some authors have used CT and MRI, which allows differentiation between a medial and lateral PTS -as we did in this study. In our study, we measured the angle between the tibial shaft and plateau, both medial and lateral. This can be converted into the PTS as described above. In our study, the mean angle between the tibial shaft and plateau was significantly higher on the lateral side. Due to the bulge of both articular surfaces, we can expect that the PTS measured in plain radiographs does not correspond with the mean value of the medial and lateral PTS. In our study, we measured the angle between the tibial shaft and plateau both medially and laterally in 50 radiographs of knees in group 3. Compared to the angle between the tibial shaft and plateau, in medial and lateral MRI scans of the same patients there were significant differences (MRI 84.2${}^{\circ }$ medial and MRI 78${}^{\circ }$, x-ray 80.2${}^{\circ }$ lateral compared to 80.2${}^{\circ }$ in x-ray).

## 5. Conclusion 

The finding that the more significantly the re-rupture-indices decrease, the higher the degree of damage is, could lead to the conclusion that patients with the anatomical configuration described above have a higher risk for ACL-injury in the future. This knowledge could be used in postoperative physiotherapy. Patients with higher risk for re-ruptures could perform proprioceptive exercises and stabilization workouts for a longer period of time to improve the intermuscular interaction. The “loading response,” i.e., the stage during walking where the leg adopts the load during walking, should be addressed. In this stage, the knee needs maximum stability [33]. The timepoint for return-to-sport could be adjusted, especially in full-contact sports. From an economical perspective, these indices could be measured in professional athletes, especially in those who already suffered from an ACL-injury. The studies discussed focus on anatomical and biomechanical aspects of the problem, in the future, stem cell based therapies may play an increased role [34].

There are some limitations, which have to be mentioned. This study focused on the configuration of the femoral condyle and its impact on ACL-tears. However, additional pathologies -like meniscus also impact ACL-stability, which is difficult to quantify. Moreover, long-axis X-ray is the golden standard to determine the PTS. In our study, we measured the PTS in MRI-scans, which is advantageous to differentiate between the medial and lateral PTS, but can be error prone due to the short image section. We used a standardized rehabilitation protocol, however in practice, compliance may vary quiet significantly and the quality and quantity of physiotherapy may also vary, due to the fact, that patients underwent physiotherapy in many different institutions. Also, the physical and sportive activity varies within our patients, which also can influence the occurance of re-rupture.

However, the present data allow defined parameters, which identify anatomical configurations of the bone, which seem to be associated with an increased risk for an ACL-(re-)rupture. These parameters are the extension facets of the medial and lateral femoral condyles, which are significantly lower in patients with ACL-re-rupture than in patients with intact ACLs. However, our findings represent one possible factor for ACL(re-)rupture, in many cases a combination of pathologies leads to (re-)tear. It is hard to objectifiable, which proportion can be lead back to the different factors.

## Funding

No funds, grants, or other support were received.

## Ethical approval

All procedures performed in the study were in accordance with the Declaration of Helsinki (1964). The study was approved by the institutional ethics committee (VT-21-1310).

## Informed consent

In accordance with the institutional ethics committee, informed consent was not necessary.

## Author contributions

Conceptualization: Lena Riemer, David Grevenstein; Methodology: David Grevenstein, Jens Dargel, Thomas Steimel, Sönke Reineck, Peter Schäferhoff; Formal analysis and investigation: David Grevenstein, Lena Riemer; Writing – original draft preparation: Lena Riemer, David Grevenstein; Writing – review and editing: Jens Dargel, Peer Eysel, David Grevenstein; Resources: Sönke Reineck, Thomas Steimel; Supervision: David Grevenstein.
